# Cranio-morphometric and aDNA corroboration of the Austronesian dispersal model in ancient Island Southeast Asia: Support from Gua Harimau, Indonesia

**DOI:** 10.1371/journal.pone.0198689

**Published:** 2018-06-22

**Authors:** Hirofumi Matsumura, Ken-ichi Shinoda, Truman Shimanjuntak, Adhi Agus Oktaviana, Sofwan Noerwidi, Harry Octavianus Sofian, Dyah Prastiningtyas, Lan Cuong Nguyen, Tsuneo Kakuda, Hideaki Kanzawa-Kiriyama, Noboru Adachi, Hsiao-chun Hung, Xuechun Fan, Xiujie Wu, Anna Willis, Marc F. Oxenham

**Affiliations:** 1 School of Health Science, Sapporo Medical University, Sapporo, Japan; 2 Department of Anthropology, National Museum of Nature and Science, Tokyo, Japan; 3 Center of Prehistory and Austronesian Studies, Jakarta, Indonesia; 4 The National Research Centre of Archaeology, Jakarta, Indonesia; 5 Institute of Archaeology, Vietnam Academy of Social Science, Hanoi, Vietnam; 6 Department of Legal Medicine, Interdisciplinary Graduate School of Medicine and Engineering, University of Yamanashi, Kofu, Japan; 7 Department of Archaeology and Natural History, Australian National University, Canberra, Australia; 8 Fujian Museum, Fuzhou, China; 9 Institute of Vertebrate Paleontology and Paleoanthropology, Chinese Academy of Sciences, Beijing, China; 10 College of Arts, Society and Education, James Cook University, Townsville, Australia; 11 School of Archeology and Anthropology, Australian National University, Canberra, Australia; Kunming Institute of Zoology, Chinese Academy of Sciences, CHINA

## Abstract

The Austronesian language is spread from Madagascar in the west, Island Southeast Asia (ISEA) in the east (e.g. the Philippines and Indonesian archipelagoes) and throughout the Pacific, as far east as Easter Island. While it seems clear that the remote ancestors of Austronesian speakers originated in Southern China, and migrated to Taiwan with the development of rice farming by c. 5500 BP and onto the northern Philippines by c. 4000 BP (the Austronesian Dispersal Hypothesis or ADH), we know very little about the origins and emergence of Austronesian speakers in the Indonesian Archipelago. Using a combination of cranial morphometric and ancient mtDNA analyses on a new dataset from Gua Hairmau, that spans the pre-Neolithic through to Metal Period (5712—5591cal BP to 1864—1719 cal BP), we rigorously test the validity of the ADH in ISEA. A morphometric analysis of 23 adult male crania, using 16 of Martin’s standard measurements, was carried out with results compared to an East and Southeast Asian dataset of 30 sample populations spanning the Late Pleistocene through to Metal Period, in addition to 39 modern samples from East and Southeast Asia, near Oceania and Australia. Further, 20 samples were analyzed for ancient mtDNA and assigned to identified haplogroups. We demonstrate that the archaeological human remains from Gua Harimau cave, Sumatra, Indonesia provide clear evidence for at least two (cranio-morphometrically defined) and perhaps even three (in the context of the ancient mtDNA results) distinct populations from two separate time periods. The results of these analyses provide substantive support for the ADH model in explaining the origins and population history of ISEA peoples.

## Introduction

The Austronesian language is spread from Madagascar in the west, Island Southeast Asia (ISEA) in the east (e.g. the Philippines and Indonesian archipelagoes) and throughout the Pacific, as far east as Easter Island. Austronesian language dispersal models have been proposed by Blust and Bellwood [1–3] and Bellwood has gone on to test these using archaeological evidence as a proxy for human movement between 5000 and 1000 years ago [1, 4–7]. The most widely recognized model that the remote ancestors of Austronesian speakers originated in Southern China, and migrated to Taiwan with the development of rice farming by c. 5500 BP and onto the northern Philippines by c. 4000 BP, is now broadly accepted [6, 7].

The subsequent Austronesian language speaking dispersals, from the Neolithic through to later Metal periods, throughout Island Southeast Asia, including Malaysia and Indonesia, and into the Pacific, referred to as the “Out of Taiwan” model or Austronesian Dispersal Hypothesis (ADH), are similarly well attested to archaeologically [8]. Notwithstanding, unlike the case in Mainland Southeast Asia (MSEA) [9, 10], human skeletal remains have not played a substantive role in human mobility debates in ISEA. The principle reason is the hitherto poor preservation of human remains from key localities in the region. For instance, Niah Cave, is the largest Neolithic mortuary site in ISEA, from which more than a hundred human skeletal remains have been reported, including the earliest dated in the region: the ‘deep skull’ [11, 12]. Unfortunately, the very poor preservation of these remains, particularly the crania, has hampered morphological analysis.

Recent excavations at the Gua Harimau cave site in southeastern Sumatra, provide an assemblage spanning the pre-Neolithic to Metal periods and an opportunity to assess the ADH model. The aim of this paper is to test the ADH using a combination of cranial morphometrics and ancient mtDNA analyses on the Gua Harimau remains. Comparisons will be made with appropriate modern and archaeological samples to better understand ancient patterns of genetic exchange and human mobility patterns in the region.

### Gua Harimau in context

**Fig 1:**
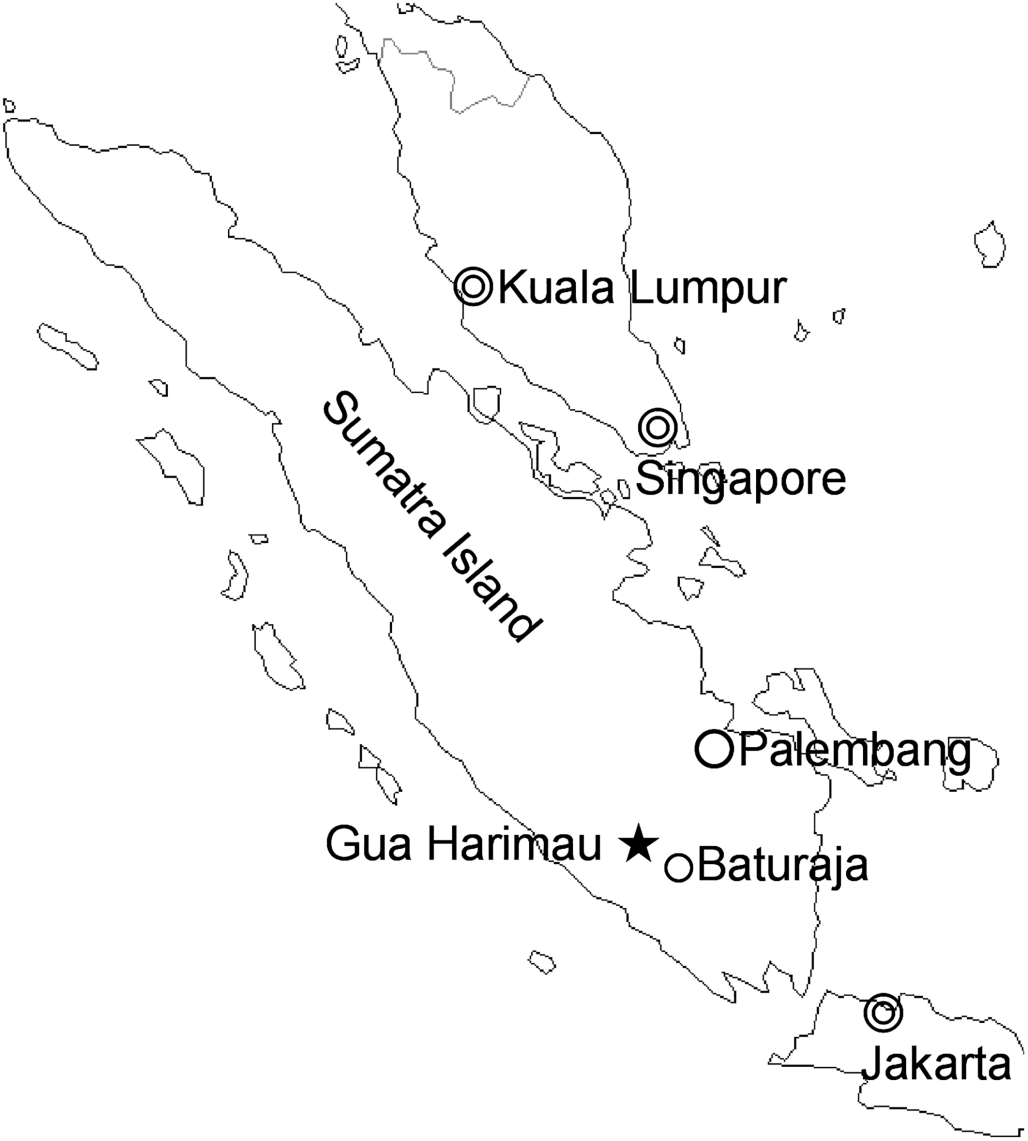
Location of Gua Harimau (star) in Southeast Sumatra, Indonesia.

**Fig 2:**
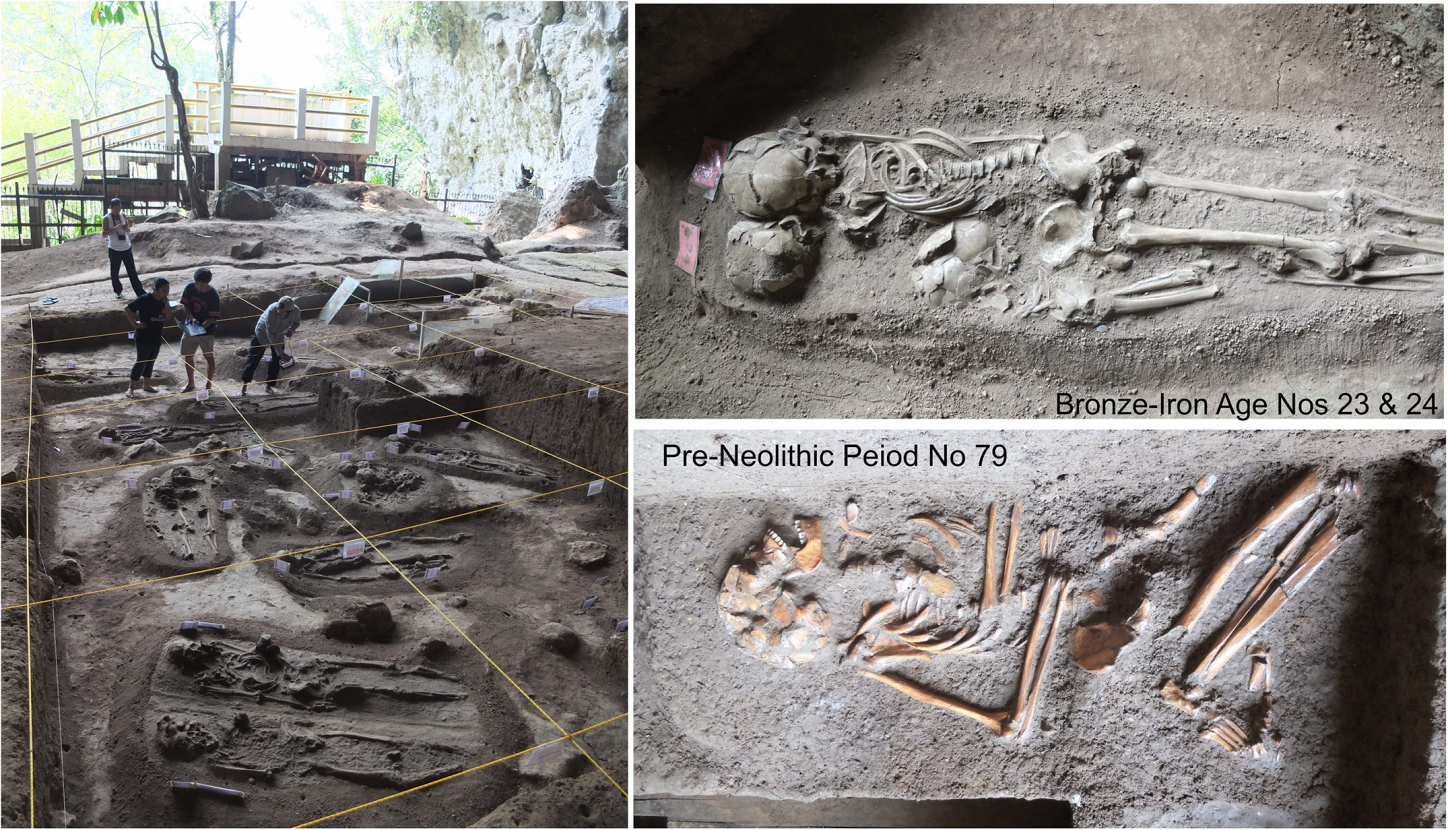
Views from Gua Harimau. Left: Metal Period (Bronze-Iron Age) extended burials (note, co-authors, from left to right: Nguyen Lan Cuong, Daya Prastingus; Hirofumi Matsumura, and Sofwan Neruwdi); Right Upper: Metal Period Burial No. 23 and 24; Right Lower: pre-Neolithic Period Burial No. 79.

The cave site of Gua Harimau is located in Padang Bindu, Oku district, in southeastern Sumatra, Indonesia (Fig 1). The cave, which formed several tens of meters above the present alluvial plain, opens towards the southeast. The width of the main entrance is c. 30m and the average horizontal depth is c. 15m. Since 2012, a substantive area of the floor of the single chambered cave has been excavated, recovering 84 individual human skeletons dating from the pre-Neolithic through to the Neolithic, Bronze and Iron Ages (Fig 2) or 5712—5591cal BP to 1864—1719 cal BP (Table 1). Some level of continuity in artefact types and floral/faunal remains from the Neolithic through to Bronze and Iron ages suggests continuity in occupation over this period of time.

**Table 1:** Results of AMS dating for human remains from the site of Gua Harimau.

ID	Lab. Code	Sample	^14^C age (BP)	±	cal BP (95.4% range)	Period
27	BTN12002	bone	1852	20	1864–1719	Metal
3	BTN12003	bone	1880	20	1879–1737	Metal
56	BTN12004	bone	1910	20	1896–1820	Metal
4	BTN12008	bone	1925	20	1923–1823	Metal
8	BTN12009	bone	1995	20	1992–1896	Metal
58	BTN12005	bone	2015	20	2003–1899	Metal
13	BTN12001	bone	2048	20	2110–1945	Metal
2	BTN12010	bone	2150	25	2304–2046	Metal
54	BTN13023	bone	2190	20	2309–2142	Metal
43	BTN13022	bone	2290	20	2352–2206	Metal
11	WK 37248	tooth dentin	2290	20	2352–2206	Metal
40	BTN12007	bone	2305	25	2356–2206	Metal
18	BTN13035	bone	2350	20	2424–2333	Metal
53	IAAA-143261	tooth dentin	2463	26	2711–2379	Neolithic
44	BTN12006	bone	2575	30	2760–2518	Neolithic
26	IAAA-170200	tooth dentin	2890	20	3136–2953	Neolithic
74	IAAA-143262	tooth dentin	4054	28	4785–4434	Pre-Neolithic
80	Beta 450669	bone	4910	30	5712–5591	Pre-Neolithic

BTN Laboratorium Batan Indonesia; WK Waitako, New Zealand. IAAA Institute of Accelerator Analysis Ltd. Japan; Beta Beta Analytic Inc. USA

^14^C ages are calibrated with OxCal v4.3, IntCal 13, Bronk Ramsey

The main feature differentiating the Metal Period burials is the presence of bronze and/or iron artefacts. Neolithic burials can be identified by way of characteristic paddle impressed and incised ceramics. Somewhat intriguingly, cord marked ceramics were also identified among some Neolithic burials. The pre-Neolithic layer, dated to between 5712–4434 cal BP, is characterized by the presence of unifacial pebble tools, similar to the almond-shaped tools often found in Hoabinhian cultural assemblages in MSEA, as well as a significant number of flakes. The deeper late Pleistocene layer, dated using charcoal to 13,055 +/- 120 ^14^C years [13] or 14,061–13,312 cal BP (95.4%) (OxCal v4.3, IntCal 13, Bronk Ramsey [14]), is characterized by numerous flakes originating from a range of materials, including obsidian. No human remains have been recovered from this basal layer.

## Materials and methods

### Cranial morphometric analysis

**Fig 3:**
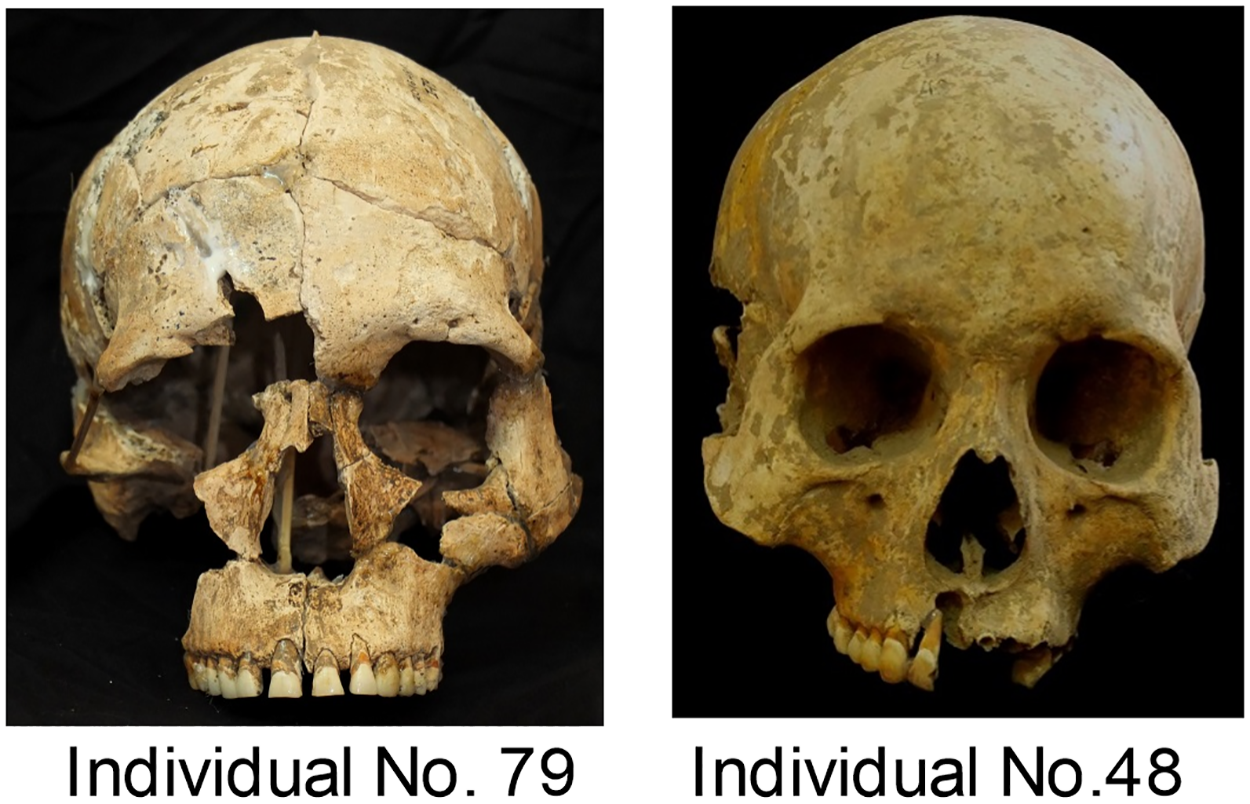
Representative pre-Neolithic (Left, individual No. 79) and Metal period (Right, individual No. 48) crania from Gua Harimau.

The excavation and analysis of Gua Harimau Cave was carried out with the permission of Dr H. Kuryana Azis (Head of the Local Government), Mr Pak Paisol (Head of the Local Tourism and Cultural Office) and Padang Bindu village. The male crania from Gua Harimau were analyzed to be consistent with standard recording protocols and generally male dominated comparative data sets available. Of the original 84 individuals, 23 were suitable for morphometric analysis and these are divided into Early and Late Gua Harimau (see representative crania in Fig 3). The Early Gua Harimau sample consists of two individuals buried in a flexed position, assigned to the pre-Neolithic period (individuals No. 74 and 79). Individual No. 74 was directly dated to between 4785–4434 cal BP. No. 79 could not be directly dated, but it was stratigraphically located between individuals No. 74 and No. 80 which was directly dated to 5712—5591cal BP. The Late Gua Harimau sample includes 20 individuals buried in a supine, extended position and one disturbed burial, the position of which could not be determined (individual No. 53), assigned to the Metal period, dated to between 2424–2333 and 1864–1719 cal BP. While three Neolithic burials could be dated (see Table 1), ranging between 3136–2953 and 2711–2379 cal BP, no Neolithic crania were sufficiently preserved to enable morphometric analysis.

The cranial data set included a subset of 16 measurements (Martin’s method numbers M1, M8, 9, M17, M43(1), M43c, M45, M46b, M46c, M48, M51, M52, M54, M55, M57, M57a), that represent the most commonly available measurements among the comparative samples. The cranio-metric affinities of the comparative samples were assessed using Q-mode correlation coefficients [15], based on the above 16 cranial measurements. The comparative archaeological cranial series are listed in Tables 2 and 3 and included a total of 64 individuals from both archaeological and contemporary contexts in East, and Southeast Asia and the Pacific. The dataset includes individuals from the late Pleistocene, early to mid-Holocene, Neolithic (defined as early farming populations [16]), and Bronze and Iron Ages through to the proto-Historic and Historic periods. Space precludes a review of each sample in the dataset, however, the references in Tables 2 and 3 provide details on the majority of samples used in this analysis.

**Table 2:** Prehistoric samples used in the present study.

Sample	Country	Period	District / Remarks / References	Data Source^a^ / Storage^b^ (n = sample size)
***Late Pleistocene*** (except Hoabinhian)				
Liujiang		Late Pleistocene	Individual (no. PA89)	[17]; M43(1),43c,46b,46c, 57,57a by H.M. (cast).
***Early Holocene Hoabinhian/Mesolithic***				
Qihedong	China	Mesolithic (c. 9,500BP)	Individual (no.3), Site in Fujian Province. [18, 19]	H.M. / IVPP
Zengpiyan	China	Mesolithic (c. 8,000BP)	Guilin, Guangxi Region [20]	[20]
Lang Gao	Vietnam	Hoabinhian	Averages of two individuals (nos. 17 and 19) [21–23]	H.M. / MHO
Lang Bon	Vietnam	Hoabinhian (c. 8,000–7,000BP)	Individual (no number) [21–23]	H.M. / MHO
Mai Da Nuoc	Vietnam	Hoabinhian (c. 8,000BP)	Individual (no. 84MDNM1) [24]	H.M. / IAH
Hoabinhian	Vietnam	Hoabinhian (c. 10,000–7,000BP)	Specimens including other fragmental remains from above 3 sites and an incomplete skull of Mai Da Dieu (no.16) [24, 25]	H.M. / IAH, MHO (n = 6)
Bac Son	Vietnam	Epi-Hoabinhian (c. 8,000–7,000BP)	Pho Binh Gia, Cua Git, Lang Cuom, and Dong Thuoc [26]	H.M. / MHO (n = 8)
Con Co Ngua	Vietnam	Mesolithic (Da But Culture, c. 6,000BP)	Sites in Than Hoa Province [27, 28] (1980's excavated series including an individual from the site of Da But [27–29])	[27]; M5,43(1),43c,46b,46c, 57, 57a by H.M./ IAH (n = 7)
Gua Cha	Malaysia	Hoabinhian (c.8,000–6,000 BP)	Individual (no. H12), Site in Kelantan Province [30]	H.M. / CAM
***Neolithic***				
Weidun	China	Neolithic (Majiabang Culture, c.7,000–5,000 BP)	Jiangsu Province [31]	[31]
Xitou	China	Neolithic (Majiabang Culture, c.7,000–5,000 BP)	Site in Fujian Province [32, 33]	H.M. / FPM (n = 3)
Tanshishan	China	Neolithic (Majiabang Culture, c.7,000–5,000 BP)	Site in Fujian Province [34, 35]	H.M. / FPM (n = 7)
Hemudu	China	Neolithic (c. 6,300 BP, Hemudu Culture)	Individual (M23), Site in Zhejiang Province, Yangzi Delta region [36]	H.M. / HEMSM
Man Bac 1	Vietnam	Late Neolithic (c. 3,800–3,500 BP)	Ninh Binh Province (immigrant group) [16, 37]	H. M. / IAH
Man Bac 2	Vietnam	Late Neolithic (c. 3,800–3,500 BP)	Ninh Binh Province (indigenous group) [16, 37]	H. M. / IAH
An Son	Vietnam	Late Neolithic (c.3,800 BP)	Long An Province [38–40]	H.M. / LAPM (n = 4)
Ban Chiang	Thailand	Neolithic-Bronze Age (c. 4,100–2,300 BP)	Site in Udon Thani Province [41, 42]	[40]; M43(1),43c,46b,46c, 57,57a by H.M. / UH, SAC (n = 15)
Khok Phanom Di	Thailand	Late Neolithic (c. 3,800–3,500 BP)	Site in Chonburi Province [43, 44]	[45] H.M. / FAD (n = 19)
Tam Hang and Tam Pong	Laos	Neolithic (Tam Hang c. 3,500 BP, Tam Pong unknown abolute date)	[42, 46, 47] (C14 date recorded in 47 was later corrected to more modern by T. Higham)	H.M. / MHO (n = 3)
Neolithic Baikal	Russia	Neolithic [48]		[49]
Jomon	Japan	Neolithic (skeletal serie used: c. 5,000–2,300 BP) [50]	from whole Japan	[51, 52]
***Bronze—Iron Age***				
Anyang	China	Yin (Shan) Period (c. 1,500–1,027 BC)	Henan Province [53]	[54]; M43(1),43c,46b,46c, 57,57a by H.M. / AST (n = 26)
Giong Ca Vo	Vietnam	Iron Age (c. 300–0 BC)	Site in Can Gio District, Ho Chi Minh City [55]	[25]; M43(1),43c,46b,46c, 57,57a by H.M. / HCHM (n = 4)
Hoa Diem	Vietnam	Iron Age (123var yr AD-243 cal yr AD (IAAA-101437)	Khanh Hoa Province [56]	H.M. / KHPM (n = 6)
Dong Son	Vietnam	Dong Son Period (c. 1,000 BC-AD 300)	Sites of Dong Son Culture [57]	[57]; M43(1),43c,46b,46c, 57,57a by H.M. / IAH, CSPH (n = 21)
Rach Rung	Vietnam	2800 BP	Site in Moc Hoa District, Long An Province [58]	H.M. / LAPM (n = 2)
Jiangnan	China	Eastern Zhou—Former Han Periods (770 BC-AD 8)	Sites in Jiangnan Province along the Lower Basin of Yangtze River [31]	[31]
Jundushan	China	Spring and Autumn Period (c. 500 BC)	Site in Yanqing Prefecture near Beijing [59]	H.M. / PKU (n = 27)
Yayoi	Japan	Yayoi Period (c. 800 BC—AD 300)	Various sites in Northern Kyushu and Yamaguchi Districts [60]	[60]

Data source^a^: H.M. = the present first author Hirofumi Matsumura.

Storage^b^: AST = Academia Sinica of the Republic of China, Taipei; BMNH = Department of Paleontology, Natural History Museum, London; CAM = Division of Biological Anthropology, University of Cambridge; CSPH = Center for Southeast Asian Prehistory, Hanoi; FAD = the Fine Arts Department, Pimai; FPM = Fujian Provincial Museum, Fujian; HCHM = Ho Chi Minh Historical Museum, Ho Chi Minh; HEMSM = Hemudu Site Museum, Hemudu; IAH = Department of Anthropology, the Institute of Archaeology, Hanoi; IVPP = Institute of Vertebrate Paleontology and Paleoanthropology, Chinese Academy of Sciences, Beijing; KHPM = Khanh Hoa Provincial Museum, Vietnam, Nha Trang; LAPM = Long An Provincial Museum, Tan An; MHO = Laboratoire d’Anthropologie Biologique, Musée de l’Homme, Paris; PKU = School of Archaeology and Museology, Peking University, Beijing; SAC = Princess Maha Chakri Sirindhorn Anthropology Centre, Bangkok; UH = Department of Anthropology, University of Hawaii, UNLV: Department of Anthropology, University of Nevada, Las Vegas

**Table 3:** Data sources of comparative modern population samples.

Population	Cranial metrics	Facial chord & subtense^a^	Remark (M = Martin's number)	Storage^b^
Aeta Negrito Philippines	H.M. (n = 11)	H.M. (n = 11)	-	MHO
Andaman Islands	[61]	H.M. (n = 5)	M9,51,55 by H.M (n = 22)	BMNH, CAM
Australian Aborigines	[47]	H.M. (n = 21)	-	BMNH
Bunun Taiwan	[62]	H.M. (n = 16)	M45,48,51,55 by H.M. (n = 23)	NTW
Cambodia	H.M. (n = 12)	H.M. (n = 12)	-	MHO
Celebes Island Indonesia	[63]	[48]	M17,45,48,51 by H.M. (n = 6)	BMNH
Hainan Island China	[61]	H.M. (n = 24)	M48,51,55 by H.M. (n = 24)	NTW
South China	H.M. (n = 7)	H.M. (n = 7)	Hong Kong	CAM
Japan	[47]	[48]	-	
Java Island Indonesia	[63]	[48]	M17,45,48,51 by H.M. (n = 20)	BMNH, CAM
Laos	[53]	H.M. (n = 10)	-	MHO
Loyalty Islands	H.M. (n = 17)	H.M. (n = 18)	-	MHO
Melanesia	[47]	[48]	Fiji, Tongans; New Hebrides; New Guinea	-
Myanmar	[63]	[48]	M17,45,48,51 by H.M. (n = 21)	BMNH
New Britain Island	H.M. (n = 20)	H.M. (n = 19)	-	CAM
New Guinea Tolai	[47]	H.M. (n = 26)	M9,48,51 by H.M. (n = 20)	USYD, CAM
Nicobar Islands	H.M. (n = 13)	H.M. (n = 9)	-	CAM
North China 1	[47]	[48]	Kiling Prov.	
North China 2	[47]	[48]	Manchuria Prov.	
Philippines	[64]	H.M. (n = 8)	-	NMP
Seman Negrito Malaysia	H.M. (n = 1)	H.M. (n = 1)	-	BMNH
South Moluccas Islands Indonesia	[63]	[48]	M17,45,48,51 by H.M. (n = 4)	-
Sumatra Island Indonesia	[63]	[48]	M17,45,48,51 by H.M. (n = 8)	BMNH, CAM
Thai	[65]	[48]	-	
Veddah Sri Lanka	H.M. (n = 2)	H.M. (n = 2)	-	CAM
Vietnam	H.M. (n = 27)	H.M. (n = 27)	-	MHO
Okhotsk Japan	[66]	[66]	AD c.400-1,000	
Hokkaido Ainu Japan	[67]	[45]		
Mongol	[67]	[45]		
Aleut, Asian Inuit, Buryat, Chukchi, Ekven, Nanay, Negidal, Nivkh, Oroch, Troitskoe, Ulch, Yakut, Yukagir	[67]	[45]	Russia	

Facial chord and subtense^a^: (M43(1) = frontal chord (FC); M43c = frontal subtense (FS); M57 = simotic chord (SC); M57a = simotic subtense (SS); M46b = zygomaxillary chord (ZC); M46c = zygomaxillary subtense (ZS); M = Martin's cranial measurment number),

Storage^b^: institutions of materials studied by H.M. (H. Matsumura) BMNH = Department of Paleontology, Natural History Museum, London; MHO = Laboratoire d’Anthropologie Biologique, Musée de l’Homme, Paris; NTW = Department of Anatomy, National Taiwan University, NMP = Department of Archaeology, National Museum of the Philippines, Manila; USYD = Department of Anatomy, University of Sydney.

To aid the interpretation of any phenotypic affinities between the samples, Neighbor Net Split tree diagrams were generated using the software package Splits Tree Version 4.0, applied to the distance (1-r) matrix of Q-mode correlation coefficients (r) [68].

### Mitochondrial DNA analysis

Tooth enamel forms a natural barrier to exogenous DNA contamination, and DNA recovered from teeth appears to lack most inhibitors of the enzymatic amplification of ancient DNA (aDNA) [69]. In addition, because recent research reveals that the temporal bone is an ideal region from which to analyze aDNA, samples were taken from both teeth and the temporal bone in this analysis [70]. In total, 20 samples (two pre-Neolithic, three Neolithic and 15 Metal Period) from well preserved teeth and temporal bones were selected for DNA analysis. A list of all samples used in this analysis are presented in Table 4 (see Results) along with their determined haplogroups.

**Table 4:** Sample used for DNA extraction and the result of the DNA analysis from the Gua Harimau site.

Lab No	Individual No.	Sample	Dating Comments	Period	Haplogroup by APLP
1	No.1	Right temporal bone	Metal Period layer	Metal	E
2	No.2	Maxilla, Right, M2	2304–2046 cal BP	Metal	N.D.
3	No.3	Mandible, Right, M1	1879–1737 cal BP	Metal	N.D.
4	No.4	Maxilla, Right, M1	1923–1823 cal BP double burial with No. 3	Metal	N.D.
5		Right temporal bone			N.D.
6	No.8	Mandible, Right, M2	1992–1896 cal BP	Metal	N.D.
7		Maxilla, Right, M3			N.D.
8	No.9	Mandible, Right, M2	Earlier layer than No. 13 (2110–1945 cal BP)	Metal	N.D.
9		Left temporal bone			B4a
10	No.10	Maxilla, Right, M1	Triple burrial with No.11 & 12	Metal	N.D.
11	No.11	Maxilla, Right, M3	2352–2206 cal BP Triple burial with No. 10 &12	Metal	N.D.
12		Left temporal bone			N.D.
13	No.12	Mandible. Left, M3	Triple burrial with No.10 & 11	Metal	N.D.
14		Left temporal bone			N9a
15	No.14	Mandible, Left, C	Same layer as No. 3 & 4 (1879–1737, 1923–1823 cal BP)	Metal	M7
15	No.19	Maxilla, Right, M3	Same layer as No. 2 (2304–2046 BP)	Metal	N.D.
17	No.21	Mandible, Left, M2	Metal Period layer	Metal	N.D.
18	No.23	Mandible, Right, M2	Triple burial with No. 24 & 25, same layer as burial No.11 (2352–2206 cal BP)	Metal	N.D.
19		Left temporal bone			Y2
20	No.24	Mandible, Right, M2	Triple burial with No. 23 & 25	Metal	N.D.
21		Right temporal bone			Y2
22	No.25	Right temporal bone	Triple burial with No. 23 & 24	Metal	N
23	No.26	Left temporal bone	3136–2953 cal BP	Neolithic	R
24	No.27	Mandible. Right, M3	1864–1719 cal BP	Metal	N.D.
25		Right temporal bone			M
26	No.36	Maxilla, Right, M3	Same layer as No.53 (2711–2379 cal BP)	Neolithic	N.D.
27		Left temporal bone			N.D.
28	No.38	Right temporal bone	Same layer as No.53 (2711–2379 cal BP)	Neolithic	R
29	No.39	Maxilla, Right, M3			N.D.
30	No.42	Right temporal bone	Same layer as No.43 (2352–2206 cal BP)	Metal	E
31	No.49	Right temporal bone	Same layer as No.58 (2003–1899 cal BP)	Metal	N.D.
32	No.57	Right temporal bone	Same layer as No.56 (1896–1820 cal BP)	Metal	R
33	No.60	Mandible, Right, M2	Earlier layer than No. 56 (1896–1820 cal BP)	Metal	B4c
34	No.74	Maxilla, Left, M3	4572–4514 cal BP	Pre Neolithic	N.D.
35	No.79	Maxilla, Right, M2	Layer between No. 74 & 80 (4434–5712 cal BP)	Pre-Neolithic	N.D.
36		Right temporal bone			N.D.

N.D. denotes Not Determined

#### Authentication methods for DNA extraction

Mitochondrial DNA (mtDNA) analyses were performed at the National Museum of Nature and Science, Tokyo, Japan, and at Yamanashi University, which have laboratories dedicated to aDNA analysis. Standard protocols were employed to avoid contamination, including the separation of pre- and post-PCR experimental areas, UV irradiation of equipment and benches, negative extraction, and PCR controls [71].

To prevent contamination from post-excavation handling, all samples were rinsed with DNA-decontamination agents (DNAaway; Molecular Bio Products, San Diego, CA, USA) or 13% bleach solution (Nacalai Tesque Inc., Kyoto, Japan), and then washed thoroughly with distilled water before drying. Next, tooth samples were encased in Exafine silicone rubber (GC, Tokyo, Japan). The tip of the root of each tooth was removed via a horizontal cut using a cutting disk, and the dentin within the dental pulp cavity was powdered and removed through the root tip using a dental drill [72]. Powdered samples were then decalcified using 0.5 M EDTA (pH 8.0) at room temperature overnight, samples were then decalcified for a further 48 hours in a fresh EDTA solution. Decalcified samples were lysed in 500 μl of Fast Lyse (Genetic ID, Fairfield, IA, USA) with 30 μl of 20 mg/ml Proteinase K at 60°C for four hours. DNA was extracted from lysate using a FAST ID DNA Extraction Kit (Genetic ID) in accordance with the protocol described by Adachi et al. [73].

#### Data analysis and genotyping of mtDNA

mtDNA SNPs were detected using the amplified product length polymorphism (APLP) method [74, 75]. This method has been applied in aDNA analyses previously and has yielded successful results [71, 76]. In this study, 81 haplogroup-diagnostic SNPs and three deletion/insertion polymorphisms, and a 9 base-pair repeat variation in the non-coding cytochrome oxidase II/tRNALys intergenic region were analyzed using the multiplex APLP method and the primer sets described by Kakuda et al. [77]. Polymorphic sites included in this analysis are known to cover most haplogroup-defining mutations found in East and Southeast Asian mtDNAs. The constitution of the PCR reaction mixture, thermal conditions, and method for separating and detecting PCR products were undertaken following Kakuda et al. [73].

In addition to APLP analysis, next-generation sequencing (NGS) technology and the mtDNA capture method were applied to individuals, whose haplogroups were tentatively determined or ambiguously identified by APLP analysis, to determine the mtDNA haplogroup or haplotype more precisely. Libraries were prepared using 8 μl of DNA extracts and using the established protocols following Shinoda or Meyer and Kircher [78, 79]. For some of the badly degraded ancient DNA, Multiplex PCR kit (QIAGEN) was used instead of AccuPrime *Pfx* kit (Life Technologies) in the first round of PCR amplification. The TreSeq DNA LT Set A or HT (Illumina) barcode for was used for indexing. Bait preparation and mtDNA enrichment for libraries were conducted following the protocol of Maricic et al. [80] and sequenced on an Illumina MiSeq platform (MiSeq Reagent Kit 150 or 300 Cycles) with 75 or 150 cycles paired-end run.

Raw sequence reads were processed following a modified protocol of Shinoda et al. [79]. After adapter trimming and merging of paired reads with AdapterRemoval v2 (—trimns—trimqualities—minquality 25—minlength 35—collapse), the merged reads were mapped to a human reference genome (hg19) using the Burrows-Wheeler Aligner (BWA) (version 0.7.8) aln option (-l 1000) [81, 82]. Cross-contaminants among samples sequenced on the same sequence run were removed using the process outlined by Kanzawa-Kiriyama et al. [83]. The reads mapped to NUMT or mitochondrial genome were retrieved and remapped to the human mitochondrial genome (revised Cambridge Reference Sequence: rCRS) with the same criteria applied when mapping to hg19. PCR duplicates were removed using Picard MarkDuplicates (version 1.119) (http://broadinstitute.github.io/picard/), and only reads with mapping quality ≥20 were retained [84]. A mpileup file (-Q 30) was compiled using SAMtools (version 1.0), calculating coverage of width and average depth. The resulting bam file was also applied to Genome Analysis Toolkit (GATK) HaplotypeCaller [85] (-stand_emit_conf 10) to call SNPs and indels [81]. The sites with low depth (<3) and high mismatch to consensus sequences (>30 percent) were masked, allowing the manual determination of the mtDNA haplogroup based on PhyloTree-Build 17 [86]. Some SNPs that were characteristic of the haplogroup but masked because of low depth were manually re-identified. We also determined the mtDNA haplogroup by using HaploGrep2 as double check of the haplotyping [87].

We investigated the degree of terminal C to T misincorporation using PMDtools and read length distribution, both of which are characteristic of ancient DNA [88]. In order to estimate contamination frequency, we used Schmutzi software (contDeam.pl—lengthDeam 40—library double) [89].

## Results

### Cranial morphometric analysis

**Fig 4:**
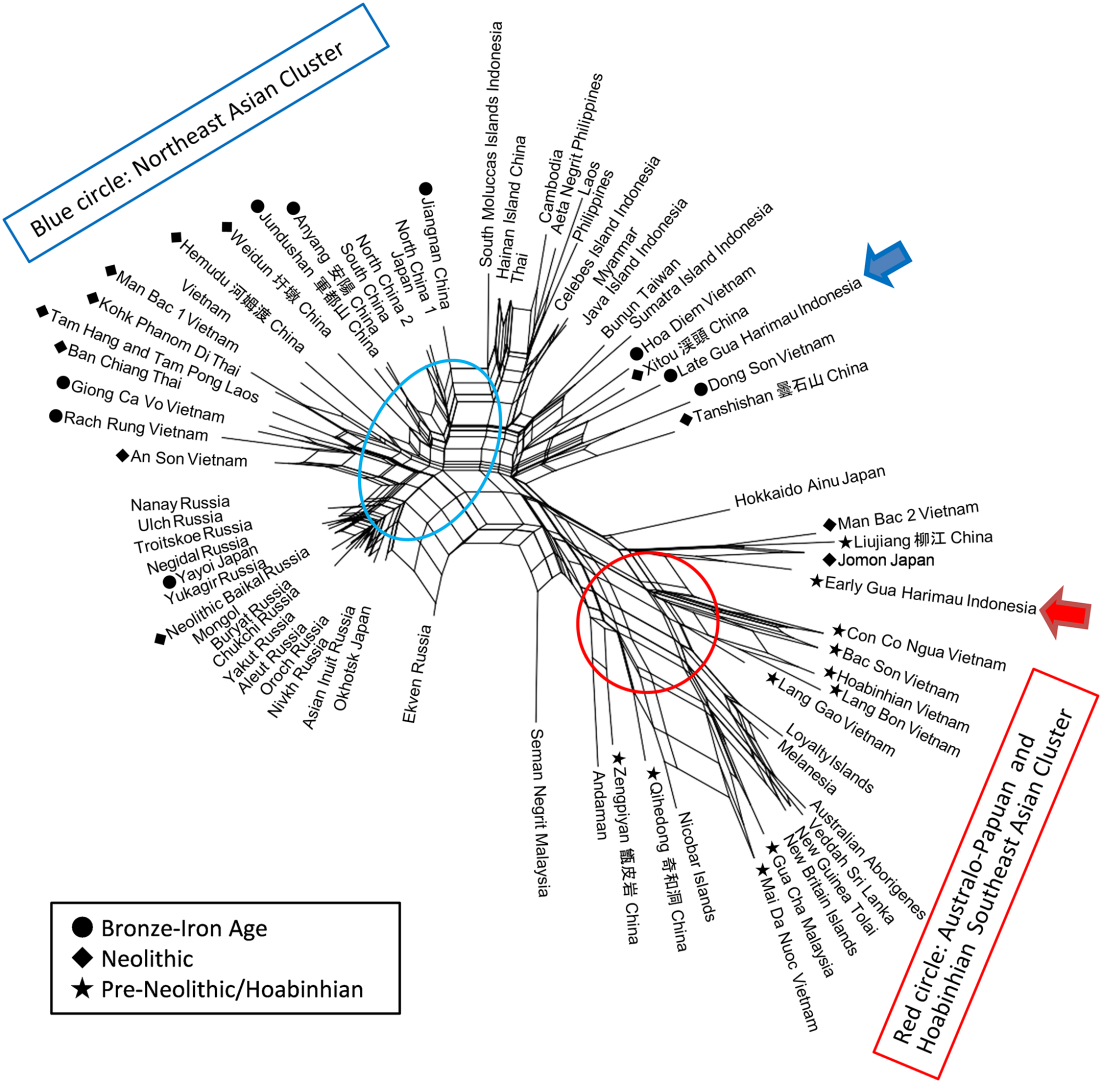
A neighbour net splits tree generated from a Q-mode correlation coefficients matrix, based on the craniometric data, comparing the archaeological and modern sample populations. The Late (pre-Neolithic) and Early Gua Harimau samples are boxed for ease of identification.

Basic statistics for the early and late Gua Harimau male series are presented in the S1 Appendix. Fig 4 presents the results of the Net Split analysis, applied to the distances of the Q-mode correlation coefficients based on 16 cranial measurements. Essentially, this unrooted network tree exhibited a straightforward dichotomization of the comparative group into two major clusters: (1) Northeast and East Asians, and several sets of Southeast Asians, ranging from the Neolithic to contemporary periods, occupy the upper left of the tree. The contemporary Southeast Asians are scattered adjacent to this cluster. (2) The Australo–Papuans, Veda of Sri Lanka, Nicobarese, Andaman Islanders, and early Holocene Southeast Asians, including Hoabinhian samples (who are morphologically quite distinct from Northeast and East Asians) form another major separate tree cluster on the lower right of the tree. It is quite interesting that Early Gua Harimau, a subset of the pre-Neolithic samples, is closely connected with the late Pleistocene and early Holocene populations, including Hoabinhian, early Bac Son, and Con Co Ngua. These samples form a mega cluster together with the Australo-Papuan and Gua Cha Malay series (Hoabinhian).

In the context of the ADH, it is notable that the Late Gua Harimau series (a Metal Period subset) exhibits close affinities with the Taiwan Formosa (Bunun), Sumatra, Moluccas, Philippines, and Celebes Island series. These current Austronesian speakers also closely cluster with Northeast Asians. Moreover, the Xitou series rom Fujian Province, one of the representative groups of the Neolithic Southern Chinese, are tightly clustered with current Austronesian speakers, as well as the Late Gua Harimau series.

The Philippine Negritos, despite possessing phenotypically different features to surrounding populations, do not show any remarkable dissimilarity to the non-Negrito Philippine samples in terms of their craniometric morphology/affinities. The Iron Age Hoa Diem sample from central Vietnam shows a close affinity to the contemporary Late Gua Harimau series, while other Vietnamese samples, including Neolithic Man Bac, An Son, Metal Age Dong Son and Giong Ca Vo more closely resemble MSEA rather than ISEA Austronesian groups.

### Mitochondrial DNA analysis

Table 4 presents the results of APLP analysis employed in the identification of mtDNA haplogroups. Of the 36 individuals considered in this analysis, we could successfully assign 13 mtDNAs of the individuals to the smallest named haplogroups and macro-haplogroups that can be identified by the APLP system employed in the present study.

In order to determine mtDNA haplogroups more precisely, from the highly fragmented DNA, we used NGS and the mtDNA capture method to investigate these tentative assigned sequences and one N.D. (not determined) sample (individual No. 79), which was the oldest of all the samples. Results of the NGS analysis are presented in Table 5. There were sufficient mtDNA reads to determine the mtDNA haplogroup or haplotype for 11 individuals. The lengths of the mtDNA fragments were very short, which is characteristic of aDNA (S2 Fig). Terminal C to T and G to A misincorporations were observed in all 11 individuals (S1 Fig). Contamination was less than two percent, so we expect that many fragments were endogenous human mtDNA. Thus, it is clear that the extracted solutions contained authentic human DNA.

**Table 5:** Results of the NGS analysis.

Individual number	Sampling	Illumina index	Library preparation method	Kit for 1st PCR	Read length of sequence	Total paired reads	Merged reads	Hits to hg 19	(%)	Remove cross contami-nation	NUMT+mtDNA	Remap to rCRS with flag 0 or 16	Removal of PCR duplicates	> = mapq20	Coverage	Average depth	Haplogroup by HaploGrep2 (quality)	Haplogroup by manual determination	Haplogroup by APLP	Contamination frequency	Additional SNPs and indels from the hg node
No. 1	Right temporal bone	505–710	[78]	Multiplex	150	872,632	580,769	262,417	45.2%	-	26,017	25,294	10,133	**10,133**	99.99%	47.2	E1a1a1a (98.16%)	**E1a1a1a**	E	0% [0–0.5%]	None
No. 4	Maxilla, Right, M1	A006	[79]	Multiplex	75	683,144	534,229	23,648	4.4%	21,201	27,995	27,933	2,729	**2,729**	96.99%	9.3	E1a1a (77.19%)	**E1a1a1**	N.D.	0% [0–1%]	C10132T (a)
		A001	[79]	AccuPrime *Pfx*	75	223,019	117,125	3,774	3.2%	2,786											
		A012	[79]	Multiplex	75	215,102	168,576	6,143	3.6%	5,596											
		A014	[79]	Multiplex	75	136,703	46,658	2,140	4.6%	1,868											
No. 4	Right temporal bone	506–711	[78]	Multiplex	150	172,000	134,839	3,400	2.5%	-	329	320	26	**26**	-	-	-	**N.D.**	N.D.	-	-
No. 9	Left temporal bone	501–710	[78]	Multiplex	150	330,343	226,354	92,824	41.0%	-	18,789	18,572	3,332	**3,332**	99.94%	12.9	R+16189 (88.15%)	B4a1a*	B4a	6.5% [4.5–8.5%]	A200G, GCA513G, T11830C, CAA16179C
No. 11	Left temporal bone	502–711	[78]	Multiplex	150	44,981	32,895	2,552	7.8%	-	576	576	60	**60**	-	-	-	**N.D.**	N.D.	-	-
No. 12	Left temporal bone	507–712	[78]	Multiplex	150	119,585	90,453	1,651	1.8%	-	276	273	67	**67**	-	-	-	**N.D.**	N9a	-	-
No. 14	Mandible, Left, C	A012	[79]	Multiplex	150	50,935	40,333	1,236	3.1%	1,071	1,104	1,097	288	**288**	71.19%	1.4	-	**(M7b1a)**	M7	-	-
		A015	[79]	Multiplex	150	71,414	29,953	747	2.5%	544											
No. 23	Left temporal bone	504–711	[78]	Multiplex	150	322,776	222,042	75,379	33.9%	-	15,460	15,238	6,688	**6,688**	99.98%	30.7	Y2a1 (92.64%)	Y2a1*	Y2	1.5% [0.5–2.5%]	None
No. 24	Right temporal bone	503–712	[78]	Multiplex	150	327,475	266,467	115,298	43.3%	-	26,055	25,809	6,775	**6,775**	99.98%	27.5	Y2a (97.25%)	Y2a1*	Y2	1.5% [0.5–2.5%]	None
No. 25	Right temporal bone	508–701	[78]	Multiplex	150	247,176	123,683	34,674	28.0%	-	6,383	6,330	1,785	**1,785**	99.40%	7.1	F1 (88.62%)	**F1a1a1**	N	0% [0–0.5%]	GCA513G
No. 26	Left temporal bone	A007	[79]	AccuPrime *Pfx*	75	2,662,710	1,825,813	1,066,688	58.4%	1,012,747	828,262	817,900	21,377	**21,376**	100.00%	109.1	R (77.01%)	R*	R	2% [1–3%]	C150T, A189G, T310TC, T450C, A3397G, G3483A, C3600A, A5484G, C6164T, A7271G, T9833C, G9966A, C10777T, G11150A, T14178C, T14311C, A15766G, T16304C
		A012	[79]	AccuPrime *Pfx*	75	2,499,051	1,745,258	904,864	51.8%	845,902											
No. 27	Right temporal bone	501–704	[78]	Multiplex	150	1,762,760	1,613,520	856,002	53.1%	-	104,366	102,986	10,381	**10,381**	99.98%	40.3	H2a2a1 (50.00%)	M*	M	0% [0–0.5%]	C151T, T152C, T310TC, GCA513G, C3817T, T4336C, T4823C, G8592A, A9285G, G11176A, C12378T, G15172A, T16229C, C16294T, T16311C
No. 36	Left temporal bone	502–705	[78]	Multiplex	150	164,461	104,406	8,240	7.9%	-	2,024	2,024	152	**152**	-	-	-	**N.D.**	N.D.	-	-
No. 38	Right temporal bone	503–706	[78]	Multiplex	150	1,279,220	1,095,331	504,191	46.0%	-	31,988	31,027	4,784	**4,784**	99.96%	19.3	B4c1b2a2 (81.63%)	B4c1b2a2*	R	0% [0–0.5%]	None
No. 42	Right temporal bone	505–702	[78]	Multiplex	150	1,111,502	803,261	420,543	52.4%	-	36,986	35,534	16,614	**16,614**	100.00%	88.8	E1a1a1a (98.22%)	**E1a1a1a**	E	0% [0–0.5%]	A302AC, T310TC
No. 49	Right temporal bone	504–707	[78]	Multiplex	150	174,450	109,241	5,247	4.8%	-	374	364	40	**40**	-	-	-	**N.D.**	N.D.	-	-
No. 57	Right temporal bone	505–708	[78]	Multiplex	150	360,438	306,470	101,159	33.0%	-	5,942	5,779	850	**850**	-	-	-	**N.D.**	R	-	-
No. 60	Mandible, Right, M2	A018	[79]	Multiplex	150	87,144	39,084	18,568	47.5%	16,288	118,606	118,533	8,273	**8,273**	99.95%	35.7	B4c1b2a2 (85.68%)	**B4c1b2a2**	B4c	0% [0–0.5%]	C146T, A302ACC, A16183C
		A002	[79]	AccuPrime *Pfx*	75	792,360	446,015	101,601	22.8%	89,515											
		A005	[78]	AccuPrime *Pfx*	150	34,517	24,477	7,639	31.2%	7,519											
		A016	[78]	AccuPrime *Pfx*	150	31,402	16,391	3,843	23.4%	3,765											
No. 79	Maxilla, Right, M2	A019	[79]	Multiplex	150	19,867	14,176	232	1.6%	144	-	-	-	-	-	-	-	**N.D.**	N.D.	-	-
No. 79	Right temporal bone	506–709	[78]	Multiplex	150	186,433	143,691	29,993	20.9%	-	1,760	1,730	420	**420**	-	-	-	**N.D.**	N.D.	-	-

N.D. denotes Not Determined

(a) Ambiguous SNP because of low depth (4) and high mismatch rate (25%)

* The haplogroup of the individual in question is ancestral, and as such was not classified into a sub-haplogroup

Complete or nearly complete mitochondrial genome sequences were determined from these 11 individuals at 7.1~109.1-fold coverage. Each haplogroup and additional SNPs and indels from the hg node are also presented in Table 5. Although individual No. 14 was classified into M7b1a, the DNA fragments were relatively long and had little misincorporation (S1, S2 and S3 Figs). In addition, length of the DNA fragments having M7b1a specific mutations are relatively longer than other reads, especially C/T or G/A damaged reads at 3 bases sequence termini, which are considered authentic DNA [90]. Therefore, we considered it to be modern human DNA contamination. For No.12, while APLP analysis assigned its mtDNA to haplogroup N9a, NGS data was insufficient for evaluating the authenticity of this mtDNA. While APLP analysis can quickly and efficiently determine a haplogroup with low cost, the NGS analysis can verify the authenticity of a haplogroup or haplotype.

## Discussion

### Pre-Austronesian indigenous populations

Before assessing the ADH, it is necessary to review the evidence for the earliest human populations in the region. The date for anatomically modern human colonization of MSEA and ISEA is attested by way of assemblages excavated in Tam Pa Ling in Laos, Niah in Malaysia, and Tabon in the Philippines, ranging from 47,000 to 30,000 years BP [91–94]. Of these, the Niah and the Tabon series were excavated from sites now occupied by Austronesian speakers, and in the context of the ADH can be seen as representative of pre-dispersal indigenous populations. However, the poor preservation of such remains limit any attempts to assess their relationship to each other or later series in the region.

It is not until the late Pleistocene to early/mid-Holocene (often referred to the Hoabinhian in MSEA), c. 23,000–8000 BP [95–98], that we have a robust sample of ostensibly pre-ADH crania. Key specimens derive from cave sites in Vietnam and Malaysia (for instance, Lang Gao, Lang Bon, Pho Binh Gia, Lang Cuom, Cua Gi, Mai Da Nuoc and Mai Da Dieu in Vietnam and Gua Cha on the central Malay Peninsula [10, 21–24, 26, 30, 96]). As shown in Fig 4, all the available Hoabinhian specimens are consistently defined as having close Australo-Papuan affinities in terms of their cranio-metrically expressed morphology. While the focus of this analysis is on male crania (see Methods), female material (e.g., Hang Cho, Gua Gunung Runtuh, and Moh Khiew) have also demonstrated remarkable cranial and dental similarities to Australian and/or Melanesian samples, suggesting a close biological affinity [99–101]. The network tree diagram (Fig 4) further indicates that some Pleistocene and early Holocene samples from China (Liujiang and Zenpiyang from Guangxi) share morphological similarities with MSEA Hoabinhian samples. Furthermore, the cranial traits characterizing these early indigenous inhabitants in the region (for instance, in northern Vietnam), were retained through the subsequent pre-Neolithic Da But Culture (c. 6700–4500 BP), clearly suggesting that such pre-agricultural foraging communities were likely direct lineal descendants of Hoabinhian foragers.

The earliest reliably dated anatomically modern humans in the region have been found in Southeast Asia, suggesting the initial colonization of the region via India, rather than north and inland through Siberia (see discussion in Buckley and Oxenham [102]). Moreover, these first colonists shared a common ancestry with the earliest settlers of continental Sahul (Australian and New Guinea). Indeed, there is a long history of scholarship [9, 10] suggesting morphological similarities, with implied genetic affinities, between Australian Aboriginals, Papuans, Melanesians and (poorly preserved) pre-Neolithic populations in Southeast Asia (e.g., Tabon in Philippines and Niah, Gua Cha, Guar Kepha, and Gua Kerbau in Malaysia). The current analysis of a more extensive cranial dataset finds further support for close affinities between early Southeast Asians, including Hoabinhian samples and Australian and Papuan-Melanesian groups, as well as the Andaman and Nicobar Indians. These observed close biological ties linking Sahul, early mainland Southeast Asia, and Eastern India, strongly suggest that the first anatomically modern human colonizers of this region migrated to the southern rim of Eurasia and then dispersed into late Pleistocene Sundaland (Southeastern Asia), including what is now ISEA. Pre-Austronesian indigenous populations may, in turn, share a common ancestry with early Hoabinhian populations in MSEA and present-day Australian Aboriginal, Papuan, and Melanesian peoples. In fact, as depicted in Fig 4, the pre-Neolithic samples from Gua Harimau (Early Gua Harimau) show a close affinity with these early settlers of MSEA and Sahul, or the first anatomically modern humans in the region.

### Austronesian dispersal

The cranio-metric analysis (see Fig 4) demonstrates a close association between the Late Gua Harimau (Metal period) and contemporary Taiwan (Bunun), Sumatra, Moluccas, Philippines, and Celebes Island samples. The morphological affinities between these series suggests a significant level of genetic interaction among neighboring inhabitants of ISEA in the past. The clustering of the Hoa Diem sample with the aforementioned series is worth discussing in more detail.

The large mortuary site at Hoa Diem, located in Khanh Hoa Province in central Vietnam, is interesting in terms of its assumed ancestry to the Chamic people of the same region. The excavation of this site has produced a large number of jar burials and associated mortuary ceramics that are strikingly similar to those from Kalanay Cave in the Philippines [52]. Similarities in material culture between the Philippines and central coastal Vietnam, as well as cranial morphometric clustering of Indonesian (Late Gua Harimau) and coastal Vietnam (Hoa Diem) populations collectively suggest substantive connections and interactions among Island populations bordering the South China Sea during the Iron Age.

Regionally, the prehistoric dispersal of Austroasiatic speakers across MSEA and Austronesian speakers throughout ISEA and the Pacific has been explicitly associated with the spread of farming during the Neolithic and subsequent early Bronze and Iron ages [1, 3, 5, 103–117]. Linguistic data indicate that Southern China and Taiwan were the origin of many of the existing language families of Southeast Asia, while archaeological evidence places the origins of Neolithic farming societies in the Yangzi and Yellow River Basins during the early Holocene, prior to their subsequent expansion into Southern China, Southeast and eastern Asia [5, 118–122].

These major Neolithic demographic transitions (NDT) in the region are often referred to in terms of the two-layer-model, whereby the first layer refers to late Pleistocene occupation of East and Southeast Asia, with the second layer being characterized by the NDT and the arrival of the ancestors of contemporary Austroasiatic (MSEA) and Austronesian (ISEA and the Pacific) speakers. Modern day Australians, Papuans and Melanesians represent the direct descendants of the first layer populations, while the descendants of the second layer include the somewhat heterogenous populations characterizing the Neolithic through to modern times.

The results from the cranial morphometric analysis in this study clearly supports the two-layer-model for both MSEA and ISEA by demonstrating close morphological associations between widely dispersed pre-NDT samples (or first layer populations) in the broader region. For instance, the early Holocene Qihedong series from Fujian Province, China, and Early Gua Harimau sample from Sumatra, Indonesia, cluster together within the Australo-Papuan group (see Fig 4). On the other hand, evidence for the spread of second layer (or NDT populations) can be identified by way of the close affinities between the Late Gua Harimau series, a number of Austronesian speaking assemblages from ISEA, and the Neolithic Southern Chinese sample from Xitou.

Turning to the genetic evidence, in disagreement with the Austronesian Dispersal Hypothesis (ADH), or Out-of-Taiwan model, is Cox and colleagues work [123, 124] which argued for a significant genetic cline across ISEA and the Pacific, ostensibly traced back to incoming populations from MSEA. Cox et al. [124] concluded that the phenotypic gradient likely reflects a mixing of two major ancestral source populations; one descended from the initial occupants of the region who were related to modern Melanesians, and the other related to Asian immigrants since the Neolithic period. Other research has also rejected the idea of large-scale demographic movement during the Neolithic, advocating for local evolutionary processes in the context of evidence for a common genetic heritage derived from the late Pleistocene colonization of Sundaland [125, 126]. As for the Austronesian expansion into mainland Southeast Asia, mtDNA analysis of Austronesian-speaking Cham individuals in central Vietnam suggests that cultural, rather than genetic, links were more a factor in this case [127]. Other DNA studies have argued that Southeast Asia was a major geographic source of East Asian populations, within which the roots of all present-day East Eurasians are historically united via a single primary wave of migration into the region [128, 129].

In this study we were able to determine mtDNA haplogroups for 11/36 samples. Three individuals (Nos. 9, 38 and 60) were identified as a subgroup of haplogroup B, a common haplogroup in ISEA that is comprised of two main clades: B4 and B5. Most of these B lineages in ISEA fall within haplogroup B4, while B5 is relatively rare. The bulk of B4 in ISEA is B4a with its major branch, B4a1, including the so-called ‘Polynesian motif’. Although Hill et al.’s [125] mtDNA analysis indicated that the dispersal of haplogroup B4a1 was triggered by postglacial flooding in the late Pleistocene or early Holocene, B4a1a has a similar distribution to that of Austronesian speakers. Gua Harimau individual No. 9 was assigned to haplogroup B4a1a, suggesting their ancestry may be Austronesian.

Arguably, lineages of haplogroup B are largely the result of a second wave of dispersal of proto-Austronesian speakers. The ancestral forms of haplogroups B4b, B4c, and B5b are found in South Chinese populations, a mainland origin, and subsequent dispersal into ISEA. The B4c haplogroup has been found in samples of ancient Negrito hair, potentially indicating a diffusion of this haplogroup from the mainland [130].

Two Gua Harimau individuals (No. 38 and 60) were classified into sub-haplogroups of B4 using whole-mtDNA sequence analysis: B4c1b2a2. Haplogroup B4c was found to have an age between 32,000 BP and 25,000 BP, with sub-haplogroup B4c1 originating between 27,000 BP and 24,000 BP, B4c1b2 to between 16,000 BP and 14,000 BP, with the origin of B4c1b2a2 dating to the Neolithic [131]. According to the DNA Database in Japan, haplogroup B4c1b2a is found in South China (Liaoning and Zhejiang provinces), as well as among aboriginal Taiwanese, the Philippines, and Indonesia. Given this demographic distribution, sub-haplogroup B4c1b2a appears to be the group associated with the Austronesian expansion during the Neolithic and/or post-Neolithic periods.

Haplogroup E is common in ISEA [125], and is frequently carried by aboriginal Taiwanese, however, it is otherwise almost absent in China and the Pacific. It has been proposed as a potential hypothetical genetic marker of Austronesian-speaking people [126]. Notwithstanding, others have attributed the origin of this haplogroup to an early Holocene population expansion originating within ISEA, which is inconsistent with the Neolithic agriculturally-driven population dispersal hypothesized in the ADH model [125, 126]. In fact, there are two major subclades, E1 and E2. Of these, E1 comprises two additional subclades, E1a and E1b, the former almost entirely restricted to Taiwan and ISEA, while the latter is found predominantly in the ISEA but absent in Taiwan.

Previously, haplogroup E itself dates to over 25,000 BP and lineages within haplogroup E have dates ranging from 6,000 BP to 16,000 BP, while a recent study based on ancient DNA calibration and Bayesian dating suggests that haplogroup E probably arose 8,136–10,933 ya (95% highest posterior density, HPD) and the majority of E lineages show a coalescence at 5–8 kya with a higher mean probability at about 6 kya [132]. According to this new time frame, Ko et al. (2014) reconstructed a history of early Austronesians arriving in Taiwan in the north ~6,000 ya, spreading rapidly to the south, and leaving Taiwan ~4,000 ya to spread throughout ISEA, Madagascar, and Oceania. Based on the demographic distribution and new time depth, E1a1a is a candidate for the presumed out-of-Taiwan dispersal. Spatial frequency distribution and diversity suggest that this haplogroup arose within ISEA, while some of its subclades subsequently spread to Taiwan [126]. This haplogroup probably evolved within the descendants of the Austronesian-speaking groups originating from Taiwan.

Relatively poor mtDNA preservation of Gua Harimau individual No. 4 (Metal period, c. 2,196–1,786 BP) makes identification of its sub-haplogroup difficult. Notwithstanding, individual No. 4’s sequences were tentatively classified as E1a1a based on diagnostic coding site changes. The greater diversity of haplogroup E in ISEA compared to Taiwan is consistent with the expansion of populations from the south [125, 126]. However, E1a1a has a lower diversity in Philippine and Sulawesi populations than it does among Taiwanese aboriginals, despite making up a larger proportion of these populations [126]. While haplogroup E may be a marker of postglacial expansion, clades within this haplogroup, such as E1a1a, possibly reflect the impact of later population events [133]. The most plausible explanation for this observation is that the diffusion of the haplogroup E1a1a in the Gua Harimau population occurred after the Neolithic expansion.

Haplogroup Y2a1 was observed in Gua Harimau individuals No. 23 and 24. This haplogroup is also common in ISEA and shared by Philippine, Taiwanese aboriginal, and other ISEA populations [133]. Y2 has a slightly higher frequency in the Philippines compared with surrounding groups. The existence of this haplogroup suggests a genetic link between ISEA and Gua Harimau populations.

The two somewhat common and widespread Southeast Asian mtDNA haplogroups are B and R9, the latter encompassing haplogroup F, with F1a being widespread in Southeast Asia. The “Early Train” hypothesis [134] claimed large scale late Pleistocene/early Holocene dispersals from MSEA into Sunda, and helps explain the distribution of F1a. Gua Harimau individual No. 25 belongs to haplogroup F1a1a1, which has been observed in high frequencies in MSEA, suggesting a link between Gua Harimau and the mainland. F1a1a1 of individual No. 25 was probably introduced by the early train, although it is still consistent with the possibility that the haplogroup entered into the Gua Harimau population by way of the Neolithic Austronesian expansion, since it is unknown whether No. 25 pre-dates the expansion. Unclassified haplogroups R* (Gua Harimau individual No. 26) and M* (Gua Harimau individual No. 27) appear unrelated to any other global lineages, are the new basal R and M haplogroups, and represent indigenous haplogroups in ISEA. Table 5 presents the complete genome substitutions of these cases. Individual No. 26 has the diagnostic polymorphisms of macrohaplogroup N (rCRS positions at 8701, 9540, 10398, 10873, and 15301), macrohaplogroup R (rCRS positions at 12705 and 16223), and 18 specific nucleotide substitutions. Individual No. 27 has the diagnostic polymorphisms of macrohaplogroup M (rCRS positions at 489, 10400, 14783 and 15043) and 15 specific nucleotides substitutions.

There are several rare ancient haplogroups within macrohaplogroup N and its sub-haplogroups R and M in ISEA. The C14 AMS dating of Gua Harimau individual No. 26 (group R) places it at c. 3000 BP, or prior to major settlement by Metal period migrants, while individual No. 27 (group M) dates to the metal period at c. 2000 BP. It seems likely that these haplogroups are relics of the original Pleistocene inhabitants of ISEA. This view is based on evidence from the persistence of mtDNA ostensibly characterizing the earliest settlers of the region. Indeed, as discussed above in the context of cranial morphometric analysis, the pre-Neolithic indigenous Gua Harimau population can potentially trace their maternal ancestry back to the first anatomically modern settlers of ISEA. It is noteworthy that the Gua Harimau gene pool consists of Austronesian (B4a1, B4c, E1a and Y2), mainland (F1a), and putative indigenous (R* and M*) forms. The mtDNA analysis is limited in estimating the composition of the three lineages making up the Gua Harimau population as well as the manner in which they genetically changed over time at the site. Nuclear genome analyses are required in order to gain further detail.

## Conclusions

The archaeological human remains from Gua Harimau cave, Sumatra, Indonesia provide evidence for at least two (cranially defined) and perhaps three (in the context of the ancient mtDNA results) distinct populations from two separate time periods, thus supporting the ADH or two-layer-model. The cranial data indicate that the pre-Neolithic occupants of (Early) Gua Harimau, who cluster with the Australo-Papuan series, were subsequently replaced by a population with close cranial affinities to present-day Austronesian speakers, including Taiwanese aboriginals, who possess Northeast Asian features to a certain extent. Further, it is apparent that the Neolithic Southern Chinese, represented by Xitou in Fujian Province, share close cranial affinities with both Austronesian speaking samples and the (Late) Gua Harimau series, supporting the view that their remote homeland was somewhere in Southern China. The results from the mtDNA is not consistent with the view (based on DNA studies of modern populations, [123, 125]) of a single origin, stretching back into the Pleistocene, for the Gua Harimau population. While the two-later-model is well supported for MSEA [9, 10, 16], this study now provides substantive support for the value of the two-layer-model in also explaining the population history of ISEA.

## Supporting information

S1 Appendix Cranial and mandibular measurements (mm) for the Gua Harimau series. (DOCX)

S1 Fig Pattern of postmortem misincorporation. C to T indicates C in reference genome and T in Gua Harimau samples, and G to A indicates G in reference genome and A in Gua Harimau samples. For No. 26, reduction of the misincorporation in 5’ end compared to 3’ end is explained by the inability of AccuPrime Pfx to bypass uracils, which is frequent in sequence termini.
(JPG)

S2 Fig Fragment size distribution of sequence reads mapped to rCRS. Only sequences having mapping quality equal or larger than 20 were used. PCR duplicates were removed.
(JPG)

S3 Fig Fragment size distribution of GH14. GH14 includes all mapped reads, and GH14 damaged includes the reads having C/T or G/A changes at 3 bases of sequence termini. White circle indicates the reads having mutations relating to haplogroup M7b1a. Those reads are relatively longer than other reads, and we considered that these are contaminants.
(JPG)
