# Supplementary material for: Cranio-morphometric and aDNA corroboration of the Austronesian dispersal model in ancient Island Southeast Asia: Support from Gua Harimau, Indonesia
Source: PLoS One. 2018 Jun 22;13(6):e0198689. doi: 10.1371/journal.pone.0198689 (PMC6014653; doi:10.1371/journal.pone.0198689)
Supplement: S1 Appendix — (DOCX) [file pone.0198689.s001.docx]

| SI Appendix. Cranial and mandibular measurements (mm) for the Gua Harimau series | | | | | | | | | | | | | | | | |  |  |  |  |  |  |  |  |  |  |  |  |  |  |  |  |
| --- | --- | --- | --- | --- | --- | --- | --- | --- | --- | --- | --- | --- | --- | --- | --- | --- | --- | --- | --- | --- | --- | --- | --- | --- | --- | --- | --- | --- | --- | --- | --- | --- |
| Individual No. | 3 | 4 | 9 | 12 | 13 | 14 | 17 | 19 | 20 | 21 | 23 | 24 | 27 | 36 | 48 | 49 | 53 | 53 | 57 | 59 | 60 | 74 | 79 | Early Gua Harimau | | | Late Gua Harimau Average | | | | | |
| Series (E=Early, L=Late) | L | L | L | L | L | L | L | L | L | L | L | L | L | L | L | L | L | L | L | L | L | E | E | Male Average | | | Male | | | Female | | |
| Sex (M=male, F=female) | F | M | M | M | M | F | M | M | F | M | M | F | F | M | F | M | M | M | F | F | M | M | M | n | M | SD | n | M | SD | n | M | SD |
| 1.Max. cranial length | - | - | - | - | 179 | - | - | 178 | 174 | - | - | - | - | 177 | 177 | 170 | 175 | 175 | 182 | 185 | 176 | 189 | 180 | 2 | 184.5 | 6.4 | 6 | 175.8 | 3.2 | 4 | 179.5 | 4.9 |
| 5.Basion-nasion length | - | - | - | - | - | - | - | 96 | - | - | - | - | - | 92 | 104 | - | 103 | 103 | - | - | 100 | - | 99 | 1 | 99.0 | - | 4 | 97.8 | 4.8 | 1 | 104.0 | - |
| 8.Max. cranial breadth | - | - | - | - | 135 | - | - | 148 | 132 | - | - | - | - | 134 | 134 | 142 | 142 | 142 | 139 | 137 | 135 | 134 | 136 | 2 | 135.0 | 1.4 | 6 | 139.3 | 5.6 | 4 | 135.5 | 3.1 |
| 9.Min. frontal breadth | 96 | - | - | - | 92 | - | - | 97 | 95 | 96 | - | - | - | 92 | 89 | 94 | 94 | 94 | 97 | 102 | 94 | 103 | 99 | 2 | 101.0 | 2.8 | 7 | 94.1 | 1.9 | 5 | 96.0 | 4.5 |
| 10.Max. frontal breadth | - | - | - | - | 112 | - | - | 118 | - | 111 | - | - | - | 111 | 113 | 108 | 118 | 118 | 123 | 120 | 111 | 113 | 121 | 2 | 117.0 | 5.7 | 7 | 112.7 | 3.8 | 3 | 118.5 | 5.3 |
| 12.Max. occipital breadth | - | - | - | - | 113 | - | - | 112 | 100 | - | - | - | - | 102 | 98 | 110 | 109 | 109 | - | 110 | 105 | 110 | 103 | 2 | 106.5 | 4.9 | 6 | 108.5 | 4.2 | 3 | 102.7 | 6.7 |
| 17.Basion-bregma height | - | - | - | - | - | - | - | 139 | - | - | - | - | - | 131 | 139 | - | 138 | 138 | - | - | 133 | - | 143 | 1 | 143.0 | - | 4 | 135.3 | 3.9 | 1 | 139.0 | - |
| 29.Frontal chord | - | - | - | - | 104 | - | - | 118 | - | - | - | - | - | 104 | 113 | - | 109 | 109 | 113 | 115 | 107 | 112 | 117 | 2 | 114.5 | 3.5 | 5 | 108.4 | 5.8 | 3 | 113.7 | 1.4 |
| 30.Parietal chord | - | - | - | - | 105 | - | - | 109 | - | - | - | - | - | 120 | 113 | - | 116 | 116 | 112 | 115 | 112 | 115 | 109 | 2 | 112.0 | 4.2 | 5 | 112.4 | 5.9 | 3 | 113.6 | 1.7 |
| 31.Occipital chord | - | - | - | - | 109 | - | - | 108 | - | - | - | - | - | 109 | 94 | - | 90 | 90 | - | - | 100 | - | - | 0 | - | - | 5 | 103.4 | 8.2 | 1 | 94.3 | - |
| 40.Basion-prosthion length | - | - | - | - | - | - | - | - | - | - | - | - | - | - | 97 | - | 100 | 100 | - | - | 93 | - | 99 | 1 | 99.0 | - | 2 | 96.5 | 4.9 | 1 | 96.7 | - |
| 43.Upper facial breadth | - | - | - | 106 | 104 | - | - | 105 | 106 | 108 | - | - | - | 104 | 99 | 106 | 104 | 104 | 102 | 109 | 107 | 117 | 114 | 2 | 115.3 | 2.4 | 8 | 105.6 | 1.5 | 4 | 103.8 | 4.1 |
| 45.Bizygomatic breadth | - | - | - | - | 138 | - | - | 136 | 145 | 136 | - | - | - | 130 | 132 | - | 136 | 136 | - | 128 | 133 | - | 142 | 1 | 142.0 | - | 6 | 134.8 | 2.8 | 3 | 134.9 | 8.9 |
| 46.Bimaxillary breadth | - | - | - | 106 | 102 | - | - | - | 98 | 103 | - | - | - | 96 | 96 | - | 100 | 100 | - | 96 | - | 98 | 104 | 2 | 101.0 | 4.2 | 5 | 101.3 | 3.8 | 3 | 96.6 | 1.5 |
| 48.Upper facial height | - | - | - | 76 | 73 | 67 | - | - | 65 | 67 | - | - | - | 65 | 69 | - | 70 | 70 | - | 72 | 70 | 69 | 70 | 2 | 69.5 | 0.7 | 6 | 70.2 | 4.0 | 4 | 68.1 | 3.0 |
| 51.Orbital breadth | 39 | 40 | - | 41 | - | - | - | - | 39 | 39 | - | 25 | 37 | 35 | 40 | - | 40 | 40 | - | 42 | 41 | 42 | 45 | 2 | 43.6 | 2.0 | 6 | 39.3 | 2.2 | 6 | 37.0 | 6.3 |
| 52.Orbital height | 35 | - | - | 38 | 33 | - | - | - | 36 | 33 | - | - | 30 | 34 | 33 | - | 34 | 34 | - | 36 | 32 | 33 | 38 | 2 | 35.5 | 3.5 | 6 | 33.9 | 2.1 | 5 | 34.1 | 2.5 |
| 54.Nasal breadth | - | - | - | 25 | 24 | 25 | - | - | 26 | 26 | 25 | 26 | - | 24 | 25 | - | 28 | 28 | - | 25 | 22 | 26 | 26 | 2 | 26.0 | 0.0 | 7 | 25.0 | 1.9 | 5 | 25.3 | 0.6 |
| 55.Nasal height | - | - | - | 54 | 55 | 48 | - | - | 49 | 48 | - | - | - | 50 | 55 | - | 55 | 55 | - | 55 | 48 | 52 | 50 | 2 | 51.0 | 1.4 | 6 | 51.6 | 3.5 | 4 | 51.7 | 3.5 |
| 60.Upper alveolar length | - | - | - | - | 61 | - | - | - | - | - | - | 54 | - | 57 | 50 | - | 55 | 55 | - | - | 59 | 59 | 60 | 2 | 59.6 | 0.9 | 4 | 58.1 | 2.5 | 2 | 52.1 | 2.6 |
| 61.Upper alveolar breadth | - | - | - | 72 | 63 | - | 67 | - | - | - | - | 65 | - | 60 | 62 | - | 66 | 66 | - | 57 | 70 | 66 | 68 | 2 | 66.8 | 1.2 | 6 | 66.3 | 4.4 | 3 | 61.2 | 4.2 |
| 43(1) Frontal chord (FC) | 102 | - | - | 95 | - | - | - | 97 | 95 | - | - | - | - | - | 91 | - | 94 | 94 | 100 | 96 | 93 | 106 | 103 | 2 | 104.5 | 2.2 | 4 | 94.6 | 1.8 | 5 | 96.7 | 4.4 |
| 43c Frontal subtense (FS) | - | - | - | 12 | - | - | - | 16.3 | 12 | - | - | - | - | - | 15 | - | 15 | 15 | - | 18 | 15 | 11 | - | 1 | 11.0 | - | 4 | 14.4 | 1.8 | 3 | 15.1 | 3.2 |
| 57 Simotic chord (SC) | 12 | 11 | - | 9.15 | - | - | - | - | - | - | - | - | - | - | 9 | - | 11 | 11 | 12 | 9.4 | 8.1 | 8.9 | 9.7 | 2 | 9.3 | 0.6 | 4 | 9.8 | 1.5 | 4 | 10.5 | 1.5 |
| 57a Simotic subtense (SS) | 4.4 | - | - | - | - | - | - | - | - | - | - | - | - | - | 2.5 | - | 4.4 | 4.4 | - | 2.8 | 2.4 | 5.3 | - | 1 | 5.3 | - | 3 | 3.8 | 1.2 | 3 | 2.9 | 0.5 |
| 46b Zygomaxillary chord (ZC) | - | - | - | 103 | - | - | - | - | 93 | - | - | - | - | - | 94 | - | 100 | 100 | - | 97 | 90 | 96 | 104 | 2 | 100.0 | 5.7 | 3 | 97.7 | 7.0 | 3 | 94.9 | 1.9 |
| 46c Zygomaxillary subtense (ZS) | - | - | - | 20.6 | - | - | - | - | 11 | - | - | - | - | - | 18 | - | 19 | 19 | - | 18 | 19 | 21 | - | 1 | 21.0 | - | 3 | 19.2 | 1.2 | 3 | 16.0 | 4.0 |
| 66.Bigonial breadth | - | - | 99 | 104 | - | - | - | - | - | - | - | - | - | - | 114 | - | - | - | - | - | - | - | - | - | - | - | 2 | 101.5 | 3.5 | 1 | 113.6 | - |
| 68 Mandibular length | - | - | 84 | 76 | - | - | - | - | - | - | - | - | - | - | 82 | - | - | - | - | - | - | - | - | - | - | - | 2 | 80.0 | 5.7 | 1 | 82.0 | - |
| 69. Symphyseal height | - | - | - | 33 | - | - | - | - | - | - | - | 30 | - | - | - | - | - | - | - | - | - | 37 | - | 1 | 37.0 | - | 1 | 33.0 | - | 1 | 30.0 | - |
| 70.Ramus height | - | - | 67 | 76 | - | - | 58 | - | - | - | - | - | - | - | 60 | - | - | - | - | - | - | 60 | - | 1 | 60.0 | - | 3 | 66.9 | 9.2 | 1 | 60.3 | - |
| 71.Ramus breadth | - | - | 43 | 37 | - | - | 36 | - | - | - | - | - | - | - | 40 | - | - | - | - | - | - | 39 | - | 1 | 39.0 | - | 3 | 38.8 | 3.7 | 1 | 40.3 | - |
