# Supplementary figures and images for: Cranio-morphometric and aDNA corroboration of the Austronesian dispersal model in ancient Island Southeast Asia: Support from Gua Harimau, Indonesia
Source: PLoS One. 2018 Jun 22;13(6):e0198689. doi: 10.1371/journal.pone.0198689 (PMC6014653; doi:10.1371/journal.pone.0198689)

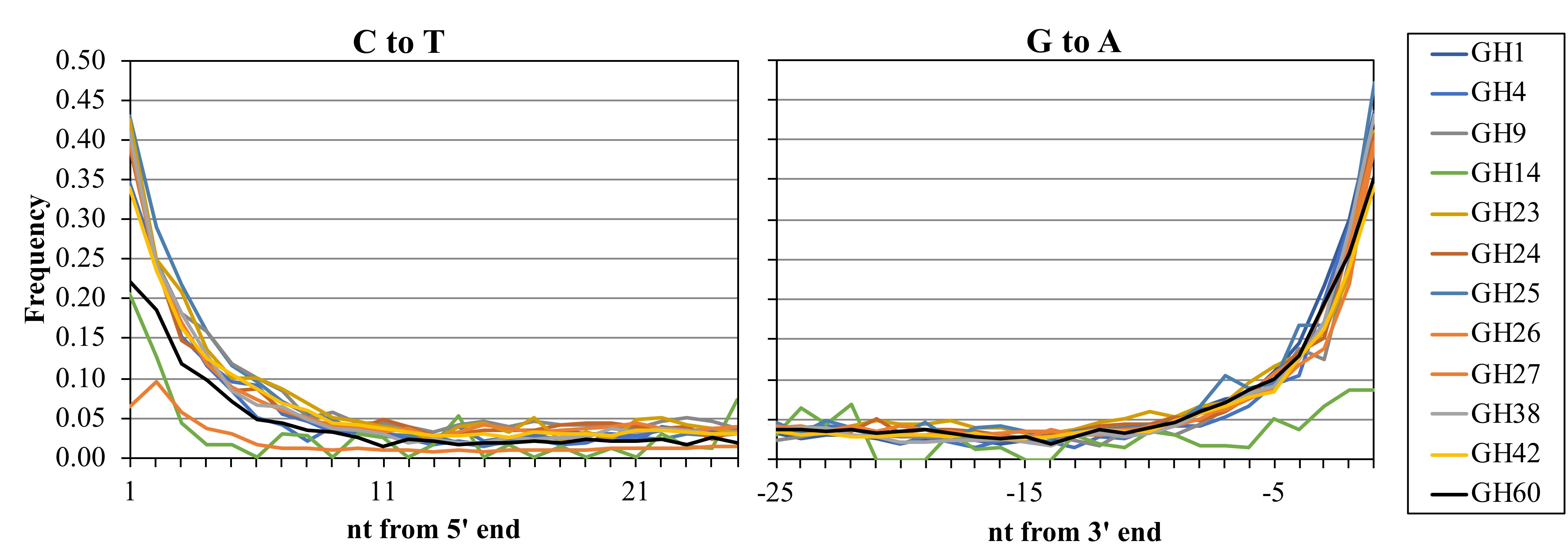

Supplement: S1 Fig — C to T indicates C in reference genome and T in Gua Harimau samples, and G to A indicates G in reference genome and A in Gua Harimau samples. For No. 26, reduction of the misincorporation in 5’ end compared to 3’ end is explained by the inability of AccuPrime Pfx to bypass uracils, which is frequent in sequence termini. (JPG) [file pone.0198689.s002.jpg]

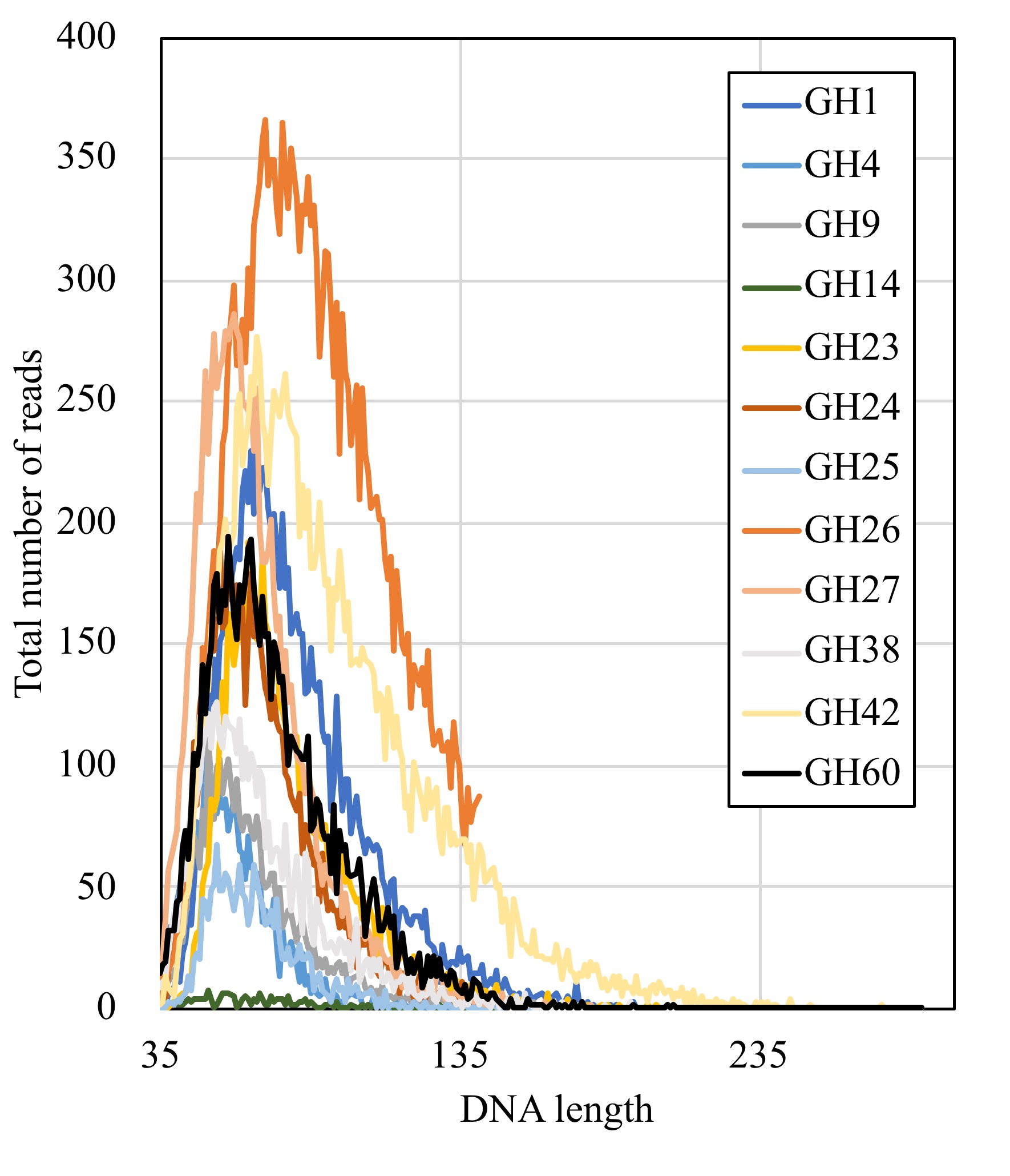

Supplement: S2 Fig — Only sequences having mapping quality equal or larger than 20 were used. PCR duplicates were removed. (JPG) [file pone.0198689.s003.jpg]

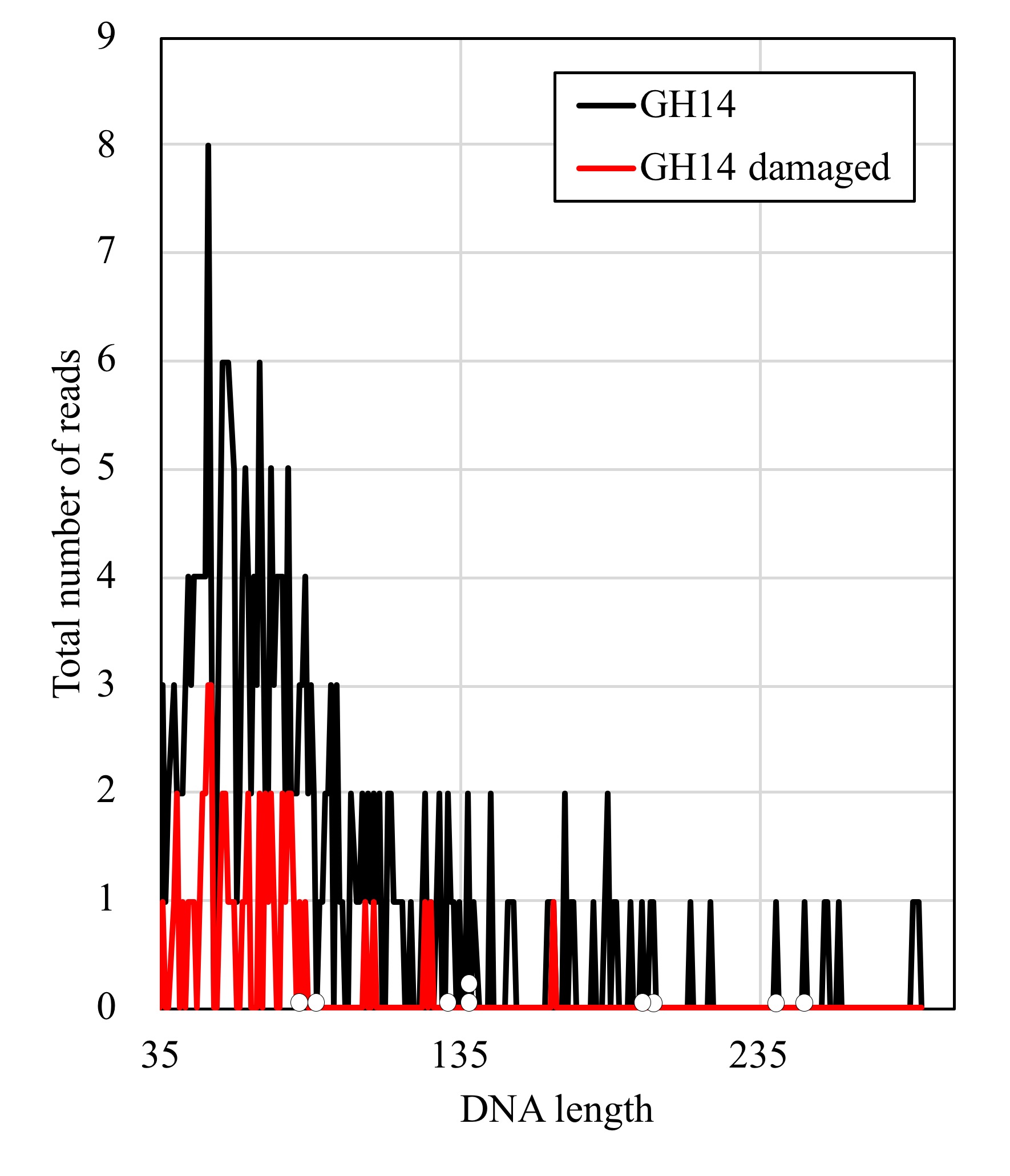

Supplement: S3 Fig — GH14 includes all mapped reads, and GH14 damaged includes the reads having C/T or G/A changes at 3 bases of sequence termini. White circle indicates the reads having mutations relating to haplogroup M7b1a. Those reads are relatively longer than other reads, and we considered that these are contaminants. (JPG) [file pone.0198689.s004.jpg]
